# A New Method of Embalming Bodies and Preserving Tissues

**Published:** 1882-09-20

**Authors:** 


**A New Method of Embalming Bodies and Preserving Tissues.**—Dr. Virodtzeff (Balsamirovanie, pp. xi, 164, St. Petersburg, 1881) recommends the following preparation as an efficient agent in the embalming of bodies and the preserving of tissues:

| | | |
|---|---|---|
| Thymol | 5 | parts. |
| Alcohol | 45 | " |
| Glycerine | 2,160 | " |
| Water | 1,081 | " |

It is cheap, innocuous, free from unpleasant odor, possesses the property of keeping the body soft, elastic, fresh and lifelike, and does not ruin instruments. Thymol is selected as being superior to other antiseptics, and glycerine is added, both on account of its own preservative qualities and to retard the evaporation of the fluid. For the preservation of tissues, the same solution is employed. If the cadaver be quite lean, or the tissues very delicate, equal parts of water and glycerine (1,620 of each) are combined with the above quantities of thymol and alcohol. To inject a body, half its weight of the fluid is necessary. A properly embalmed cadaver may be preserved indefinitely under ordinary circumstances, gradually shrinking and mummifying without putrefaction. Specimens are either to be injected with, or macerated in this fluid. Maceration must not be too prolonged; the appearance of the specimen should act as a guide. The part, after having been thoroughly cleansed with water, and prepared, may then be exposed for months to the air without losing its consistency, form and color. Permanent specimens may be enclosed in a hermetically sealed glass vessel containing a little of the same solution. Dr. Peabody has used this preserving fluid, with excellent results, in the New York Hospital Museum.—*London Medical Record, April 15.*

